# Case report: A *CLCN1* complex variant mutation in exon 15 in a mixed-breed dog with hereditary myotonia

**DOI:** 10.3389/fvets.2024.1485454

**Published:** 2024-11-04

**Authors:** Gabriel Utida Eguchi, Mariana Isa Poci Palumbo, Fabrício Moreira Cerri, Roberta Martins Basso, José Paes de Oliveira-Filho, Silvana Marques Caramalac, Alexandre Secorun Borges

**Affiliations:** ^1^Faculty of Veterinary Medicine and Animal Science, Federal University of Mato Grosso do Sul (UFMS), Campo Grande, MS, Brazil; ^2^Department of Veterinary Clinical Science, School of Veterinary Medicine and Animal Science, São Paulo State University (Unesp), Botucatu, SP, Brazil

**Keywords:** chloride channel, neuromuscular disorder, hereditary disease, electroneuromyography, congenital myotonia

## Abstract

At 4 months of age, a male dog was presented with a complaint of a stiff gait following a startle response. Neurological examination revealed no deficits, but clinical myotonia was easily induced upon requesting the patient to jump. Additionally, myotonia of the upper lip muscles was observed upon manipulation. Hereditary myotonia was suspected, and electromyography confirmed the presence of myotonic potentials. Genetic testing of the myotonic patient identified a complex of mutations, including c.[1636_1639 delins AACGGG] and c.[1644 A>T], both located in exon 15 of the *CLCN1* gene leading to the formation of a premature stop codon. Genetic investigations of the mother and four littermates revealed that, except for one littermate who was wild type, all others carried a copy of the mutated gene. To the best of the authors' knowledge, these mutations have not been previously reported.

## Introduction

Myotonia, defined as a delayed relaxation of muscles after contraction, is the prominent sign of hereditary myotonia (1). In animals, non-dystrophic hereditary myotonia associated with abnormal chloride channel 1 has been previously described in many species, including goats (2), dogs (3–15), cats (16–18), pigs (19), and buffaloes (20). These species have been confirmed to be affected. In dogs, it was previously associated with purebred dogs (3–11). Only one study illustrates the genetic mutation associated with hereditary myotonia in a family of mixed-breed dogs (12).

The knowledge of the mutations involved in the *CLCN1* gene could prevent the propagation of the defect, consequently reducing the incidence of dogs suffering from the condition. *CLCN1* gene mutations exhibit complex heterogeneity with different mutations found across various dog lineages (5–8, 10–12). As more mutations are discovered, more comprehensive genetic screening for suspected dogs can be developed.

Here, we describe a recessive form of hereditary myotonia in a mixed-breed dog including the clinical presentation, electrophysiological abnormalities, and genetic characterization of a novel complex exon 15 mutation of the *CLCN1* gene, along with the genetic investigation of its relatives.

## Case presentation

A 4-month-old male dog was referred with the main complaint of a stiff gait when startled or during any sudden movement initiation. This transient motor difficulty had been present since the first months of life. Otherwise, there were no other disclosed abnormalities, and the patient was reported as a normal puppy regarding growth and behavior. During the clinical evaluation, general physical parameters and neurological examination were all within normal limits. The dog had generalized muscular hypertrophy. Myotonia was easily induced, most prominently when the patient was requested to jump high steps. Additionally, due to the dog's defensive behavior, picking him up caused him to growl, and upper lip myotonia was also observed. The “warm-up phenomenon” (improvement in muscle stiffness and the ease of movement that occurs after repeated use of the affected muscles) was also present (Supplementary Video 1). The owners did not perceive the myotonic episodes as having a significant impact on the animal's overall quality of life. They noted that during the episodes, the patient might be in discomfort, but no signs of vocalization or pain behavior were described.

Considering the clinical myotonia, warm-up phenomenon, muscle hypertrophy, and absence of weakness, non-dystrophic hereditary myotonia associated with abnormal chloride channel 1 was suspected. Therefore, electromyography (EMG) was performed, and whole blood was sent for genetic investigation. Additional blood tests, including a complete blood count, creatinine, urea, alanine aminotransferase, alkaline phosphatase, total protein, and creatine kinase enzyme, were also conducted.

Blood samples were also collected from the mother and four littermates for genetic analysis. Unfortunately, no neurological examination was possible for these animals. However, no similar clinical signs were reported, and based on videos, photos, and a general physical examination performed by a generalist veterinarian at the time of blood collection, no abnormalities were found.

## Diagnostic assessment

EMG was performed (Neurotec Neuromap^®^ EQPE041) without any anesthetic medications. The band-pass filter was set between 30 and 10,000 Hz, and display settings were configured to 100 μV/division sensitivity and 10 ms/division sweep speed. The electrode was a concentric needle (Neurotecnologia^®^ D039035408 40 mm, 28 G), and the grounding was a surface electrode with an alligator clip placed on the inguinal skin.

**Figure 1:**
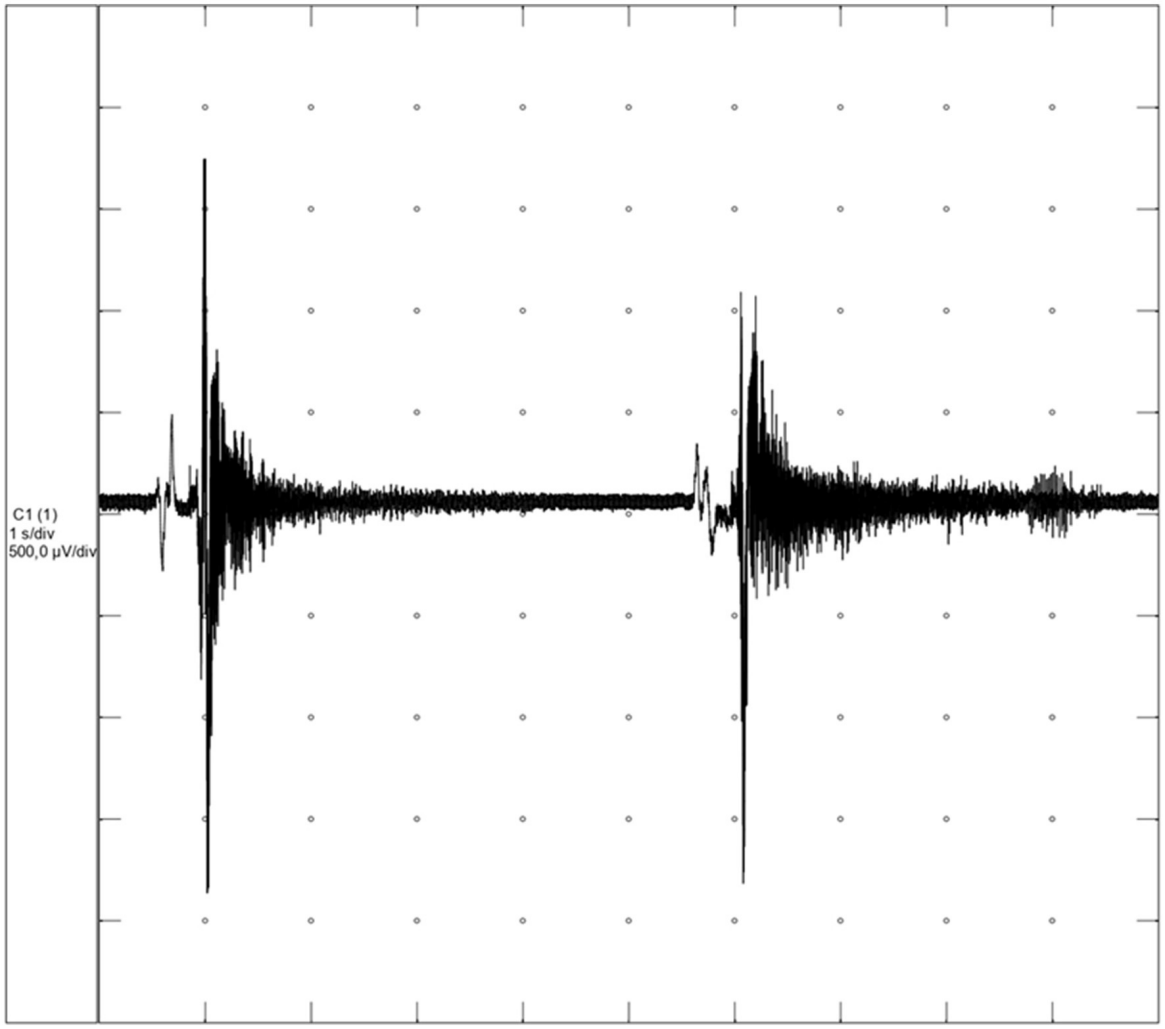
Myotonic discharges from the dog with hereditary myotonia (Neurotec Neuromap^®^ EQPE041; 500 μV/division sensitivity and 1 s/division).

Myotonic potentials were present and myotonia was also characterized by its “wax and wane” sound in the tibialis cranialis, gastrocnemius, biceps femoris, and extensor carpi radialis muscles (Figure 1; Supplementary Video 2). Ancillary blood analysis showed no abnormalities.

Blood samples obtained from the myotonic dog (dog 1), its mother (dog 2), littermates (dogs 3–6), and an unrelated wild-type dog (dog 7, sourced from the DNA bank of the Veterinary Molecular Biology Laboratory of the Department of Veterinary Clinical Science of the FMVZ-Unesp) underwent genomic DNA extraction and purification using the DNeasy Blood & Tissue kit (Qiagen) following the manufacturer's instructions. DNA concentration was then measured using spectrophotometry (Nanodrop^®^ 2000—Thermo Scientific™).

The primers used to amplify the 23 exons (coding sequence) of the canine *CLCN1* gene (Supplementary Table 1), along with the PCR conditions and thermocycling, were previously described (12). The amplicons were analyzed by 1.5% agarose gel electrophoresis, purified with magnetic beads, and sequenced via Sanger sequencing. The electropherograms were examined using Geneious Prime^®^ 2019.1.3 software. The obtained sequences were compared to the reference of the *CLCN1* gene from Canis lupus familiaris (GenBank NP_001003124.1).

**Figure 2:**
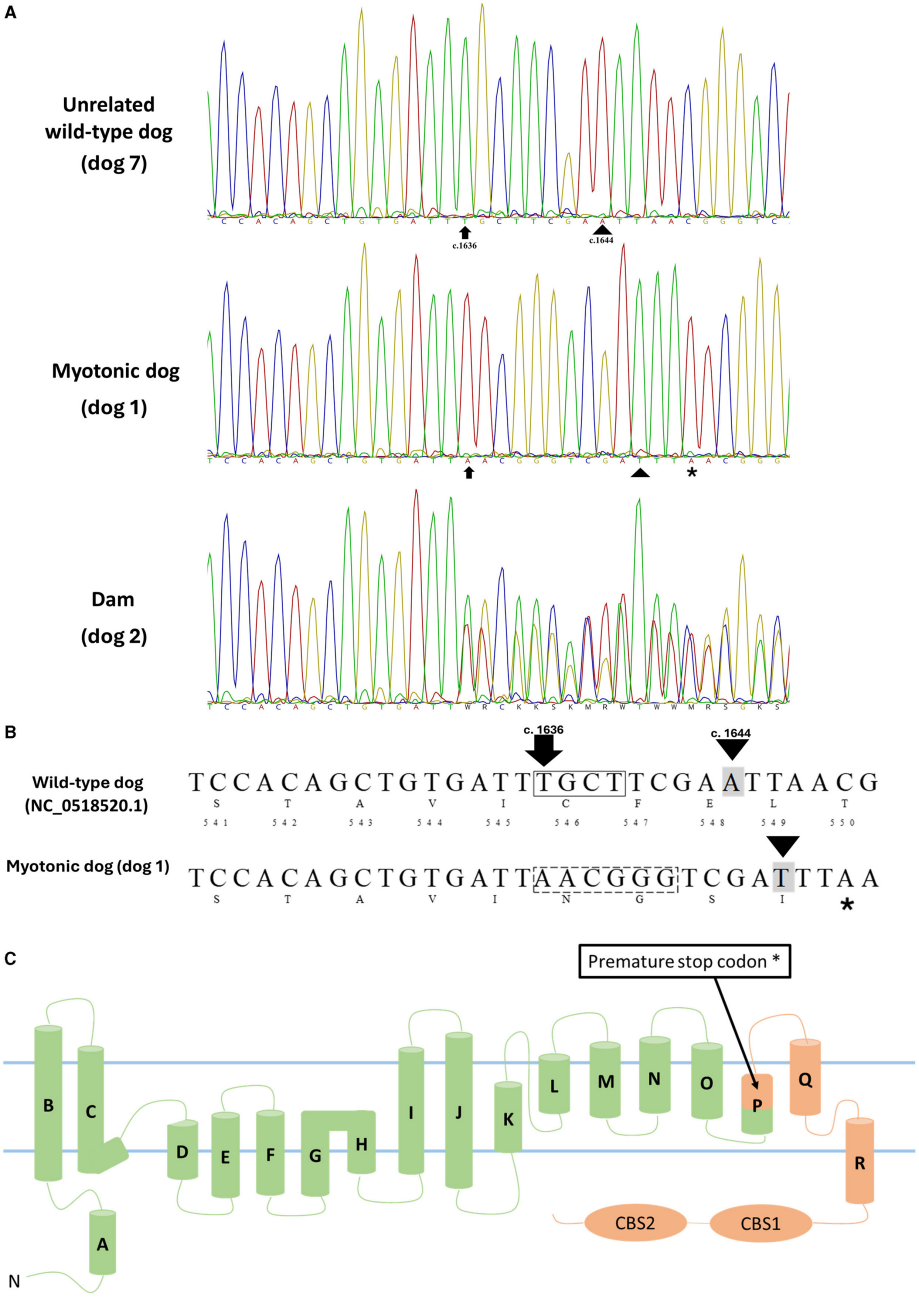
**(A)** Partial capillary sequencing chromatogram for exon 15 of *CLCN1* in an unrelated wild-type dog (dog 7), a myotonic dog (dog 1), and the dam (dog 2). The arrow indicates the start of the mutation (c.1636-1639delinsAACGGG), and the arrowhead indicates the second observed mutation (c.1644 A>T). **(B)** Schematic representation of nucleotide and amino acid sequences of the wild-type dog (top, NC_0518520.1) and the myotonic dog (bottom) with the corresponding nucleotide numbers and amino acids. The box representing four nucleotides in the wild-type sequence indicates the amino acids deleted in the myotonic animal (c.1636-1639del TGCT), while the hatched (- - -) box in the myotonic dog sequence (AACGGG) results from the insertion of 6 nucleotides (c.1636_1639delins AACGGG). The described complex mutation results in a frameshift and the formation of a premature stop codon. Nucleotide highlighted in gray indicates the observed substitution (c.1644 A>T). *Indicates a premature stop codon (TAA - c.1646-1648). **(C)** Membrane topology model of the human skeletal muscle chloride channel monomer, ClC-1 [modified from Brenes et al. (25)], representing the premature stop codon formation and compromising the structure of α-helices P (encompassing amino acids 541-551), Q and R as well as the CBS1 and 2 domains in addition to the P-Q and Q-R loops and the C-terminal, all of which play key roles in the proper functioning of this channel. In green are the structures that are preserved, and in orange are the structures that are absent in the dog with myotonia.

The alignment of sequences from the myotonic dog (dog 1), its mother (dog 2), littermates (dogs 3–6), and a wild-type dog (dog 7), along with the GenBank reference sequence (NP_001003124.1), revealed a complex of mutations c.[1636_1639delinsAACGGG] and c.[1644A>T], both in exon 15. This second mutation, c.[1644A>T], resulted in the modification of the amino acid sequence p.Glu548Asp. The chloride channel in the mutated animal has the initial 545 amino acids preserved, followed by the generation of four different amino acids (546–549). The variant complex c.[1636_1639delinsAACGGG] caused the formation of a premature stop codon in exon 15 (positions c.1646-1648). This results in a truncated protein with ~400 fewer amino acids compared to the normal CLC protein (NP_001003124.1), which has 976 amino acids in the dog (Figure 2).

Dog 4 had no alterations in the region of the observed mutation (wild type). Dogs 3, 5, and 6 each presented a copy of the mutated gene. The mother (dog 2) also showed the presence of a copy of the mutated sequence. Dogs 2, 3, 5, and 6 were considered heterozygous for the described mutations.

No treatment was instituted as the condition didn't seem to impact the patient's quality of life. Nonetheless, owner education was emphasized as a crucial aspect of managing this condition. Educating the owners about the nature of clinical myotonia is important because the episodes can cause psychological distress for some people who may mistakenly believe the dog is in active pain during these events. Additionally, owners were advised to remove all affected relatives from breeding programs to prevent the propagation of the genetic mutation.

## Outcome and follow-up

Two years after the initial evaluation, the patient showed no worsening of the condition in terms of frequency and severity. The dog exhibited normal growth and behavior, and despite some visible generalized muscle hypertrophy (Supplementary Video 1), no other clinical abnormalities were found.

## Discussion

Delayed relaxation of skeletal muscles after onset of movements or startle, muscle hypertrophy, and the “warm-up” phenomenon are highly indicative of hereditary myotonia related to chloride channel 1 abnormalities. The “warm-up” phenomenon serves as a valuable clinical tool in diagnostic criteria for hereditary myotonia (HC), distinguishing myotonic events from paradoxical myotonia, where muscle rigidity typically worsens with exercise (12, 21). Myotonia in domestic animals can also occur with sodium channel abnormalities, but these are usually associated with weakness, which was not observed in this dog.

While myotonia caused by mutations in *CLCN1* has been well-documented in purebred dogs (3–11), reports and genetic investigations in mixed-breed dogs remain limited but have been described (12). To the best of our investigation, no pure-breed ancestors could be identified in our patient.

Clinical myotonia was confirmed through EMG evaluation, which detected myotonic discharges [frequencies may range from 20 to 150 Hz, potential amplitudes range from 10 to 1,000 mV and last 500 ms or longer (22, 23)] resulting in a characteristic sound and distinguishes them from cases of pseudomyotonia, where clinical myotonia is present without myotonic potentials (1, 21). A limitation of this study is the lack of histological analysis of the musculature, as the biopsy was not authorized by the owner. This analysis is important to rule out cases of dystrophic myotonia, which was not the initial suspicion for this dog. In cases of hereditary myotonia, the muscle is usually only characterized by variation in the diameter of the fibers (15, 16).

Mutations in the *CLCN1* gene responsible for hereditary myotonia have been documented in various dog breeds, including Miniature Schnauzers (6), Australian Cattle Dogs (8), Labrador Retrievers (10), American Bulldogs (11). However, in the dog with myotonia from the present study, none of the previously reported mutations in the *CLCN1* gene associated with myotonia in dogs or other domestic animals were identified. Only one study has reported a mutation in the *CLCN1* gene in a family of mixed-breed dogs, and that mutation was a complex variant in exon 6, different from the variant found in our study, which is located in exon 15 (12). Several mutations have been described in humans in exon 15, but not at the same position as the complex variant found in our study (3, 14, 24). The complex mutation observed in this patient resulting in a premature stop codon affected the structure of α-helices P (encompassing amino acids 541–551), Q (555–571), and R (576–585), as well as the 2 intracellular C-terminal cystathionin-β-synthase (CBS) segments (609–876), in addition to the P-Q and Q-R and the C-terminal loops, all of which play key roles in the proper functioning of *CLCN1*. This importance is underscored by ~110 mutations located in this region that have been reported in humans, where amino acid alterations have led to congenital myotonia (25). Further supporting the significance of these α-helices (P, Q, and R) and CBS domains, congenital myotonia has also been described in animals, with point mutations in this region reported in a horse (26), cat (16), dogs (8–10), and goats (27). To highlight the importance of this region of the *CLCN1* gene in cases of myotonia in animals, we can mention goats, which were the first domesticated species described with myotonia and the responsible mutation is a single nucleotide change that results in the substitution of proline for a conserved alanine residue in the CBS (27). Therefore, it is reasonable to consider that, even in the absence of patch-clamp studies, the truncated *CLCN1* (~400 aa shorter than the normal chloride channel 1) observed in the dog from the present study is responsible for the clinical myotonia. Considering the distribution of findings in the studied family and the sequencing results, it is concluded that this is an autosomal recessive form of the disease, similar to all other cases of hereditary myotonia associated with chloride channel mutations described in domestic animals to date.

Despite being a disease that typically does not severely compromise the quality of life of affected dogs, myotonia can still cause some limitations and predispose patients to accidental falls. In some cases, myotonic episodes may be frequent enough to hinder normal daily activities and require treatment attempts (10). Hereditary myotonia does not seem to reduce the life expectancy of affected individuals. Patients are often diagnosed as adults (7, 8, 10, 12) and typically experience no severe decline in quality of life or developmental issues. The patient described in our study had a follow-up period of 2 years, during which no deterioration in condition was observed. Nevertheless, from an owner's perspective, these episodes may resemble completely different physiological conditions, such as cramping syndromes, and thus owners may extrapolate the discomfort from any muscle cramp they have experienced themselves. Therefore, owner education is crucial regarding the nature of this condition and any available treatment options. When treatment is chosen, details such as type of medication, dosage, and expected outcomes have been previously reported.

Moreover, it is essential not to overlook owner education regarding the hereditary nature of this disease, and preventing mating and breeding of affected individuals should be strongly advised.

## Data Availability

The original contributions presented in the study are included in the article/Supplementary material, further inquiries can be directed to the corresponding author.

## Supplementary material

Supplementary Video 1 Dog with hereditary myotonia at first presentation with 4 months old and at 2-years follow-up; clinical myotonia episodes and “warm-up” phenomenon are observed. Note the muscle hypertrophy and upper lip myotonia (2-years).

Supplementary Video 2 EMG evaluation of the dog with hereditary myotonia (Neurotec Neuromap^®^ EQPE041; band pass filter, 30 to 10,000 Hz; display settings, 100 μV/division sensitivity and 10 ms/division sweep speed; sixty Hz filter “on”). Note myotonic discharges that wax and wane and characteristic sound.

Supplementary Table 1 Primers for PCR amplification.
