# Supplementary material for: Case report: A CLCN1 complex variant mutation in exon 15 in a mixed-breed dog with hereditary myotonia
Source: Front Vet Sci. 2024 Nov 4;11:1485454. doi: 10.3389/fvets.2024.1485454 (PMC11571544; doi:10.3389/fvets.2024.1485454)
Supplement: Supplementary Table 1 — Primers for PCR amplification. [file Table_1.doc]

**Supplementary Table 1. Primers for PCR amplification**.

| **Primer^a^** | **Direction** | **Sequence (5′-3′)** | **Product (bp)** |
| --- | --- | --- | --- |
| CLCN1_Exon 1 | Forward | AGATCAGATGTGGCAGTGG | 404 |
| CLCN1_Exon 1 | Reverse | CCCAATCCATCACACAATTACC |
| CLCN1_Exon 2-3 | Forward | GTCTATGTGCTTCCCTCAGTC | 729 |
| CLCN1_Exon 2-3 | Reverse | TCCTAGGTAGGAACTCGAGAAA |  |
| CLCN1_Exon 4-5 | Forward | TCCCTGTGTGCAATGAGAAC | 696 |
| CLCN1_Exon 4-5 | Reverse | TCCCTTCCCGGGTATTTACT |  |
| CLCN1_Exon 6 | Forward | CCTGAAACGCTGGCAATTT | 456 |
| CLCN1_Exon 6 | Reverse | CACAGATCCAGAAGGGATGG |  |
| CLCN1_Exon 7 | Forward | TTTGGCCTTTCGCTGTGA | 445 |
| CLCN1_Exon 7 | Reverse | GCTTAGTATATCACCCGCACATAG |  |
| CLCN1_Exon 8-9 | Forward | GCATCAGCAAGTGTATGGTTTAG | 821 |
| CLCN1_Exon 8-9 | Reverse | CCACTTCCTGGACCTGTTC |  |
| CLCN1_Exon 9-10 | Forward | TGAATTGCTGGAGCAGGAG | 673 |
| CLCN1_Exon 9-10 | Reverse | ATTTCATTGCAGGCTTCCTAAAG |  |
| CLCN1_Exon 11-12 | Forward | GGTGGCAAGAACATTCTATGTTTA | 663 |
| CLCN1_Exon 11-12 | Reverse | TGAAGTTGTACCCTCTCCTACT |  |
| CLCN1_Exon 13-14 | Forward | TAGCTACTCCAGGGTTTCTG | 668 |
| CLCN1_Exon 13-14 | Reverse | AAGAAACTACAGCTCAAAGACA |  |
| CLCN1_Exon 15 | Forward | GCTAAAGGACATAGAATGAGATCAA | 445 |
| CLCN1_Exon 15 | Reverse | TCAACCTCAGAACCTCCCA |  |
| CLCN1_Exon 16 | Forward | GATGGAATGCGTGGCAAAC | 368 |
| CLCN1_Exon 16 | Reverse | TAAGGAACATAGGCTGGGTATC |  |
| CLCN1_Exon 17 | Forward | ATCCTGGAAGATGTTTGAAATGAG | 428 |
| CLCN1_Exon 17 | Reverse | GGTCTCCTTGACTTTCTCAGTG |  |
| CLCN1_Exon 18 | Forward | GGGTAAATGGATGGAGACAGA | 368 |
| CLCN1_Exon 18 | Reverse | CCAACTCCTTGGGACAGTAATA |  |
| CLCN1_Exon 19-20 | Forward | TCATTTCCTCCCAGGACAGATA | 494 |
| CLCN1_Exon 19-20 | Reverse | GCAATGTCTTCATCACACAGAAC |  |
| CLCN1_Exon 21-23 | Forward | CTGCAGGTCTGAGGTCATAG | 425 |
| CLCN1_Exon 21-23 | Reverse | GGGACCACCCAGATGTTAAA |  |
| CLCN1_Exon 23 | Forward | TGTGCGAATCTGCTCTTCTC | 531 |
| CLCN1_Exon 23 | Reverse | GGTCTCCAACTTCAGGACATAC |  |

^a^Described by Chimenes et al., 2023
